# Pharmacoeconomic evaluation of isavuconazole, posaconazole, and voriconazole for the treatment of invasive mold diseases in hematological patients: initial therapy prior to pathogen differential diagnosis in China

**DOI:** 10.3389/fpubh.2023.1292162

**Published:** 2023-12-19

**Authors:** Guangxin Han, Qing Xu, Qianzhou Lv, Xiaoyu Li, Xiaoping Shi

**Affiliations:** ^1^Department of Pharmacy, Zhongshan Hospital, Fudan University, Shanghai, China; ^2^Department of Clinical Pharmacy, The First Affiliated Hospital of Guangdong Pharmaceutical University, Guangzhou, China

**Keywords:** isavuconazole, posaconazole, voriconazole, invasive aspergillosis, mucormycosis, cost-effectiveness

## Abstract

**Background:**

Invasive mold diseases (IMD) is associated with high mortality and a substantial economic burden. For high-risk patients, fever drive or diagnostic drive therapy is usually initiated prior to the differential diagnosis of the pathogen. This study evaluated the cost-effectiveness of isavuconazole, posaconazole, vs. voriconazole in the treatment of IMD from the perspective of the Chinese healthcare system, informing healthcare decision-making and resource allocation.

**Methods:**

A decision analytic model was constructed using TreeAge Pro 2011 software to evaluate the cost-effectiveness of the entire disease course. We assumed that the prevalence of mucormycosis in the patients entering the model was 7.8%. Efficacy, cost, adverse events, and other data included in the model were mainly derived from clinical studies, published literature, and publicly available databases. The primary outcomes of the model output were total cost, quality-adjusted life years (QALYs), life years (Lys), and incremental cost-effectiveness ratio (ICER). The willing-to-pay (WTP) threshold was defined as one to three times China's GDP per capita in 2022. One-way sensitivity analysis and probability sensitivity analysis were used to determine the robustness of the model. At the same time, the cost-effectiveness of three triazole antifungal agents under a broader range of mucormycosis prevalence, when voriconazole was covered by medical insurance reimbursement, and after the price reduction of posaconazole was discussed.

**Results:**

Compared with voriconazole, isavuconazole provided an additional 0.38 Lys (9.29 vs. 8.91 LYs) and 0.31 QALYs (7.62 vs. 7.31 QALYs); ICER was $15,702.46/QALY, well-below the WTP threshold ($38,223/QALY). However, posaconazole did not provide a significant economic advantage over voriconazole (9.40 vs. 9.36 Lys; 7.71 vs. 7.68 QALYs; ICER $64,466.57/QALY). One-way sensitivity analysis found that ICER was highly sensitive to the mortality of patients with invasive *aspergillus* infection. In the probabilistic sensitivity analysis, when the WTP threshold was $38,223/QALY, the probability of isavuconazole being cost-effective was 72.9%. The scenario analysis results indicated that posaconazole would become cost-effective when the price was reduced by 15% or the prevalence of mucormycosis was 14%.

**Conclusions:**

Isavuconazole represents a cost-effective initial option for treating IMD in high-risk hematological patients prior to the differential diagnosis of pathogens. It will also be economical when a 15% reduction in posaconazole cost is achieved.

## 1 Introduction

Invasive mold diseases (IMD) frequently affect immunocompromised individuals with hematological malignancies and hematopoietic stem cell transplantation. The common pathogens are *Aspergillus*, and *Mucorales* species (1). While the advancements in modern medical technology have significantly improved the survival rates and lifespans of these patients, they have also led to an increased incidence of IMD (2). The prevalence of invasive *aspergillus* (IA) varies between 0.94 and 14% among China's immunocompromised individuals (3). In Europe, there are ~60,000 annual cases of IA, whereas estimates suggest that there are over 160,000 cases of IA reported each year in China (4). IMD imposes a heavy burden on both patients and healthcare systems. A retrospective study of invasive pulmonary aspergillosis (IPA) revealed higher rates of mechanical ventilation (43.3 vs. 5%), longer hospital stays (45.8 vs. 18.4 days), and increased mortality rates (43.3 vs. 11.4%) among IPA cases compared to non-IPA patients (5). Mucormycosis, another IMD, also exerts a significant economic impact on society and individuals due to its lengthy treatment duration, high cost, and limited availability of safe and effective treatment options. According to a study conducted in China, the mortality rate of mucormycosis exceeds 40%, and the economic burden is estimated to be 3–10 times the country's annual disposable income per capita (6).

Diagnosing IA and mucormycosis in a timely manner can be challenging due to their similar clinical and radiological manifestations. Co-infections can further complicate the differential diagnosis process (7, 8). Typically, the treatment for IMD is initiated based on patient risk factors, clinical and radiological presentations before the causative agent is identified. However, the gold standard for fungal diagnosis is isolation the causative fungus from appropriate tissues and sterile samples, which presents a considerable difficulty and can result in misdiagnosis or underdiagnosis (9).

Voriconazole is the primary recommended treatment agent for IA according to the guidelines from the Infectious Diseases Society of America (IDSA), the European Society for Clinical Microbiology and Infectious Diseases (ESCMID), and the European Confederation of Medical Mycology (ECMM) (10, 11). However, it frequently leads to liver dysfunction, and its clinical utility is limited due to its inhibition of cytochrome P450 isoenzymes and the presence of sulfobutylether-beta-cyclodextrin in the intravenous formulation. Recently, a phase 3, randomized, controlled, non-inferiority trial evaluated the efficacy and safety of posaconazole for treating IA (12). Given its effectiveness, it was approved by the United States Food and Drug Administration in June 2021 for the treatment of IA. Isavuconazole is another antifungal drug used as a primary or alternative treatment agent for IA (10, 11). In comparation to voriconazole, isavuconazole has similar efficacy but significantly lower rates of drug-related adverse events, such as hepatotoxicity and visual impairment (42 vs. 60%, *p* < 0.001) (13). The rate of permanent discontinuation due to drug-related adverse events is also lower for isavuconazole than for voriconazole (8% vs. 14%) (13). Notably, in addition to their anti-*aspergillus* activity, both posaconazole and isavuconazole have shown activity against *mucorales*, whereas voriconazole has not. Consequently, the global guideline for the diagnosis and management of mucormycosis, published by the ECMM, recommends posaconazole and isavuconazole as first-line treatments for mucormycosis by Cornely et al. (14).

Voriconazole has long served as the standard treatment for IA. Nonetheless, with the introduction of isavuconazole and posaconazole, there are now more clinical treatment options available for IA. Hence, it is imperative to determine the most cost-effective treatment choices to optimize the allocation of medical resources and alleviate the financial burden on patients. Studies conducted in the United States and some European countries have shown that isavuconazole represents a cost-effective treatment option for IA or suspected IA when compared to voriconazole (15–18). However, there is currently no existing report on the cost-effectiveness of isavuconazole and posaconazole vs. voriconazole in China. To address this gap, an economic model was developed to explore the cost-effectiveness of using voriconazole, isavuconazole, and posaconazole in the treatment of hematological patients with suspected IMD when the differential diagnosis between IA and mucormycosis is uncertain at the start of treatment.

## 2 Methods

### 2.1 Model structure

**Figure 1:**
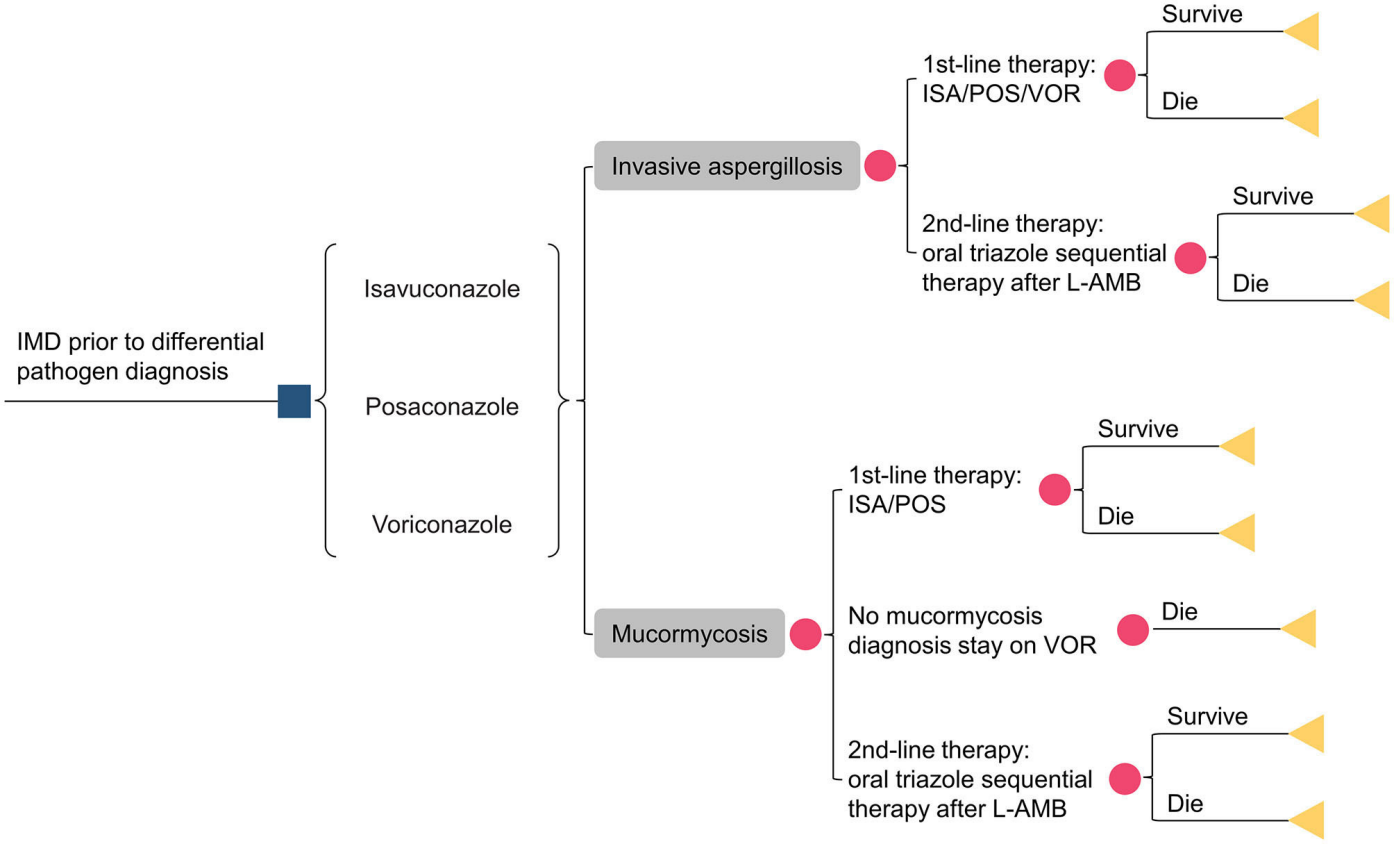
Schematic representation of the decision analysis model. IMD, invasive mold diseases; ISA, isavuconazole; POS, posaconazole; VOR, voriconazole; L-AMB, liposomal amphotericin-B.

A decision analytic model (Figure 1) was adopted from a healthcare system perspective to evaluate the cost-effectiveness of isavuconazole, posaconazole, and voriconazole for treating IMD. The model was based on the published literature (15, 16, 18) and used TreeAge Pro 2011 (TreeAge Software Inc., Williamstown, MA, USA), incorporating China-specific costs and resource utilization.

Assuming 1,000 patients with a suspected IMD entered the model, antifungal therapy was initiated before obtaining pathogen information. Patients were assigned to the following treatment sequences: isavuconazole, posaconazole, and voriconazole. Only 50% of patients had their pathogens identified, with *aspergillus* accounting for 92.2% of the cases and *mucorales* accounting for 7.8%. Given the absence of domestic data, the pathogen detection rate was referenced from previous studies (15, 18). Depending on clinical responsiveness, drug tolerance, and other factors, patients could switch to second-line treatment with liposomal amphotericin B (L-AmB). As voriconazole had no activity against *mucorales*, patients initially treated with voriconazole switched to L-AmB therapy if *mucorales* were identified during treatment. Otherwise, voriconazole therapy was maintained until the patient's demise. The sequential regimen consisted of oral triazoles. Two additional triazoles, other than the initial one, were administrated orally at a 50/50% ratio. However, for isavuconazole (posaconazole) -treated patients with mucormycosis, only oral posaconazole (isavuconazole) sequential L-AmB was allowed.

### 2.2 Model inputs and data sources

#### 2.2.1 Clinical data

The proportion of switching to second-line treatment and all-cause mortality were derived from several large clinical studies (12, 13, 19) and literature (20, 21). Since there were no head-to-head clinical studies between posaconazole and isavuconazole, *in vitro* drug sensitivity tests showed that both drugs had similar good activity against *mucorales* (22), it was assumed that the data of isavuconazole were also applicable to posaconazole for mucormycosis.

#### 2.2.2 Treatment regimen and duration

Patients entering the model were assumed to have normal liver and kidney function or not require dose adjustment. Of all patients, 75% received intravenous therapy initially and then switched to oral treatment, while 25% received oral medicine at the outset (15, 16, 18). The daily loading and maintenance doses of isavuconazole, posaconazole, and voriconazole were 600/200 mg, 600/300 mg, and 800/400 mg, respectively, whether administrated orally or intravenously. The dose of L-AmB was administrated at 5 mg/kg, and the mean body weight (60 kg) of Chinese patients with hematologic malignancies or hematopoietic stem cell transplantation was used to calculate the total daily dose of L-AmB (23, 24).

The treatment duration in the model was 67 days (intravenous 9 days) for invasive *aspergillus* (IA) (12) and 149 days (intravenous 15.5 days) for mucormycosis (19). Referring to the published literature, isavuconazole, posaconazole, and voriconazole were thought to have the same course of treatment (15, 16, 18). On average, patients who did not respond to first-line therapy changed their regimen on day 21 because most no-responders switched treatment between days 1 and 42 (13, 19). However, patients treated with voriconazole who were later diagnosed with mucormycosis changed their regimen on day 11 (25); those who did not receive a diagnosis of mucormycosis continued to be treated as IA for 67 days (intravenous 9 days). After the failure of first-line treatment, L-AmB took 14.5 days to treat IA (26) and 27.2 days to treat mucormycosis based on previous literature (19).

The average length of hospital stay (LOS) for first-line treatment of IA and mucormycosis was 19.7 days (13) and 19.3 days (19), respectively. Due to a lack of data for second-line treatment, we hypothesized an extended LOS to (19.7 + 21) and (27.2 + 21) days for treatment of IA and mucormycosis, respectively, to meet the required course of treatment and the necessary LOS. Clinical experts also endorsed this hypothesis.

#### 2.2.3 Costs

The unit costs of isavuconazole (Cresemba^®^), posaconazole (Noxafil^®^), and voriconazole (Vfend^®^) were available from a tertiary care general hospital with 2,000 beds. Furthermore, Fengkesong^®^ was the commonly used L-AmB in Chinese hospitals, so we adopted its price. The model also incorporated additional resources utilized beyond the cost of antifungal drugs, such as the costs of laboratory testing, microbiological detection, galactomannan antigen determination, imaging examination, hospitalization cost (cost for bed utilization, nursing fees, etc.), and outpatient follow-up cost. Considering the pharmacokinetics advantages of isavuconazole, the actual situation of Chinese hospitals, and the recommendations of the 2017 ESCMID-ECMM-ERS guideline (11), therapeutic drug monitoring was only conducted for posaconazole and voriconazole. Severe hepatotoxicity was considered the only adverse event that required intensive therapy and affected the cost. The incidence of severe hepatotoxicity was obtained from phase 3 clinical trials (12, 13) and publicly available databases. An artificial liver support molecular adsorbent recirculating system will be used to treat liver failure, and the cost was a one-time fee obtained from a study in China (27).

#### 2.2.4 Utility and life expectancy

Since the key clinical outcomes of the model were derived from several clinical studies mainly targeting acute myeloid leukemia patients (13, 19), and the underlying disease will impact the quality of life and life expectancy for survivors of IMD, a lifetime horizon was chosen to capture the long-term effects and costs of the three compared drugs. A quality of life utility value of 0.82 (28), a life expectancy of 17 years (29), and a discount rate of 3% were applied to discount the costs and health impacts. All input data for the decision-tree model were shown in Table 1.

**Table 1:** Input data used for the decision-tree model.

**Parameter**	**Isavuconazole**	**Posaconazole**	**Voriconazole**	2nd-line treatment^*^
**Epidemiology inputs**				
**Prevalence of invasive aspergillosis**	92.2%			
**Prevalence of mucormycosis**	7.8% (15, 18)			
**Clinical inputs**				
**Proportion switching to 2nd-line treatment** ^**#**^				
Invasive aspergillosis	47.7% (13)	42.7% (12)	45.3%/38.3%	–
Mucormycosis	35.1% (19)	35.1%	100.0%	–
**All-cause mortality**				
Invasive aspergillosis	20.0% (13)	19.0% (12)	23.0%/19.0%	65.0%
Mucormycosis	43.2% (19)	43.2%	100.0%	82.9%
**Incidence of severe hepatotoxicity**	1.2% (13)	3.1% (12)	2.8%	–
**Duration of treatment (days)**				
Invasive aspergillosis	67 (IV: 9.0) (12)			14.5 (26)
Mucormycosis	149 (IV: 15.5) (19)		–	27.2 (19)
**Duration prior to switching 2nd-line therapy (days)**	21 (IV: 9.0) (13, 19), except voriconazole for mucormycosis: 11 (IV: 9.0) (25)			
**Length of hospital stay (days)**				
Invasive aspergillosis	19.7 (13)			19.7 + 21
Mucormycosis	19.3 (19)		27.2 + 11	27.2 + 21
**Days from therapy to death (days)**				
Invasive aspergillosis	37			–
Mucormycosis	28			–
**Economic inputs**				
**Unit of drug costs ($)**				
IV	336.26	282.32	138.29	18.75
Oral	89.95	39.62	39.00	–
**Cost of treatment other than drugs ($)**				
Single laboratory test cost	102.67			
Single microbiological detection cost	86.18			
Single GM test	22.29			
Single imaging examination cost	29.72			
Other hospitalization costs per day	21.99			
Single outpatient follow-up cost	3.71			
Therapeutic drug monitoring	22.29			
The cost of DILI's treatment	3,982.17			
**Utility (quality of life)**	0.82			

^*^Liposomal amphotericin B is 2-line treatment drug.

^#^The proportion switching to 2nd-line treatment was calculated by the numbers of patients who discontinued 1st-line treatment minus the number of patients who died during treatment.

IV, intravenous; GM, galactomannan; DILI, drug-induced liver injury.

### 2.3 Cost-effectiveness outcomes and model analysis

The model calculated quality-adjusted life years (QALYs), life years (LYs), and costs in each sequence. The incremental cost-effectiveness ratios (ICER) were represented by the ratio of cost to QALY. ICERs were compared to the willingness-to-pay (WTP) threshold of 3 times China's GDP per capita in 2022 (3 × $12,741) to assess cost-effectiveness.

In order to evaluate the robustness of the model, one-way sensitivity analysis was performed to examine the impact of varying the parameter on ICER. The following parameters were modified within a certain range: cost parameters, utility parameters, and duration of treatment were varied by ±25% of the base-case values; probability parameters were varied by ±10% of the base-case values. The upper and lower bounds of parameters within the range of 0–1 were restricted within their respective boundaries. The tornado diagrams displayed which parameters were the key drivers of the results.

Probabilistic sensitivity analysis (PSA) using 10,000 Monte Carlo iterations were performed to account for uncertainty in the model inputs and estimate the likelihood of different outcomes. Probability and utility parameters (bounded by 0 and 1) were assigned a beta distribution, while costs and treatment duration (due to positive values and bounded at 0) were assigned a gamma distribution. The standard error for some parameters was assumed to equal 10–25% of the mean because of lacking information on their variability. The results of PSA were presented by the cost-effectiveness acceptability curve, which showed the probability of compared drugs being cost-effective over a range of WTP threshold.

Scenario analysis was used to examine the model's results under different scenarios or hypothetical situations. Given the absence of domestic data on *mucorales*, the impact of this parameter on ICER was tested by using both a higher (14%) and a lower (2%) pathogen detection rate, respectively. Voriconazole, a well-established classic drug, is covered by health insurance in China, while the other two drugs are not. Eligible patients only bear a minimal account (10%) for voriconazole. We introduced this reduced cost of voriconazole into the model to assess its cost-effectiveness when compared to other triazole drugs, for which patients need to cover the full cost. Finally, considering the possibility of price decreases after the loss of exclusivity, we assessed the influence of changing drug price on the results.

## 3 Results

### 3.1 Base-case analysis

The outcomes and costs over a lifetime horizon with isavuconazole, posaconazole, and voriconazole were shown in Table 2. The ICERs were calculated to voriconazole, which was regarded as the standard antifungal therapy. Isavuconazole demonstrated greater efficacy (+0.38 LYs and +0.31 QALYs) than voriconazole, albeit at a higher cost (+$4,857.71). An ICER of $15,702.46 for each extra QALY obtained was just only 23% higher than China's GDP per capita in 2022.

**Table 2:** Base case cost-effectiveness results.

**Parameters**	**Isavuconazole**			**Voriconazole**			**Incremental value**
	**IA**	**IM**	**Combined**	**IA**	**IM**	**Combined**	
Cost ($)	20,794.19	1,814.21	22,608.39	16,285.18	1,465.50	17,750.68	4,857.71
LYs	9.24	0.05	9.29	8.91	0.01	8.91	0.38
QALYs	7.58	0.04	7.62	7.30	0.01	7.31	0.31
ICER ($/QALY)							15,702.46
**Parameters**	**Posaconazole**			**Voriconazole**			**Incremental value**
	**IA**	**IM**	**Combined**	**IA**	**IM**	**Combined**	
Cost ($)	17,159.57	1,443.36	18,602.93	15,025.51	1,465.50	16,491.01	2,111.92
LYs	9.35	0.05	9.40	9.35	0.01	9.36	0.04
QALYs	7.67	0.04	7.71	7.67	0.01	7.68	0.03
ICER ($/QALY)							64,466.57

IA, invasive aspergillosis; IM, invasive mucormycosis; LYs, life years; QALYs, quality-adjusted life years; ICER, incremental cost-effectiveness ratio.

In contrast, posaconazole did not provide a significant cost-effectiveness advantage over voriconazole. The treatment cost of posaconazole was higher (+$2,111.92), but it only had a slight therapeutic advantage (+0.04 LYs and +0.03 QALYs). It resulted in an ICER of $64,466.57 per additional QALY gained, which was well over 3 times GDP per capita.

### 3.2 One-way sensitivity analysis

**Figure 2:**
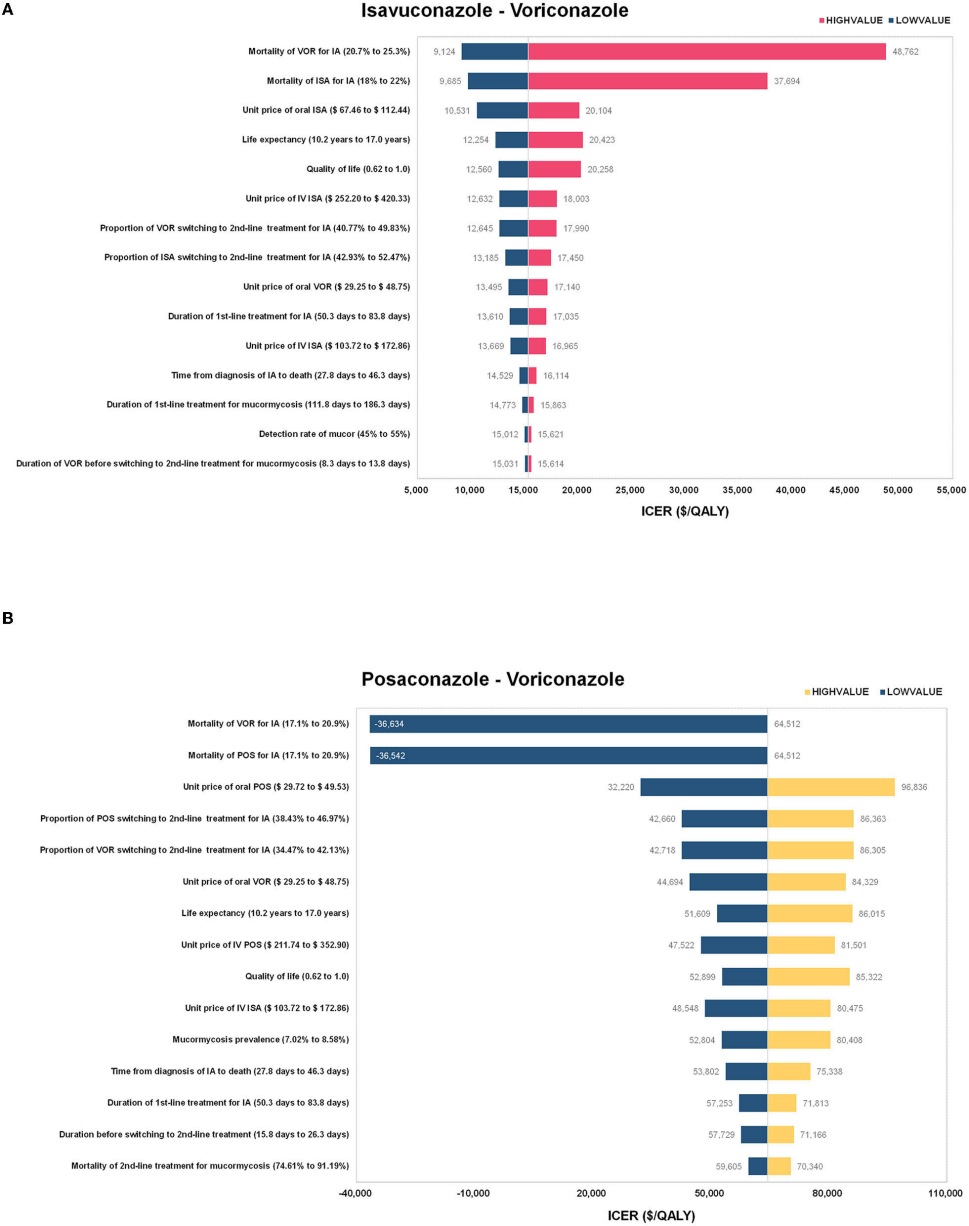
The results of the one-way sensitivity analysis for **(A)** isavuconazole versus voriconazole and **(B)** posaconazole versus voriconazole. ISA, isavuconazole; POS, posaconazole; VOR, voriconazole; IA, invasive aspergillosis; IV, intravenous; ICER, incremental cost-effectiveness ratio; QALY, quality-adjusted life year.

In the one-way sensitivity analysis, a total of 35 parameters were tested, and the 12 parameters that greatly impacted the model results were presented in Figure 2. It was found that ICER was most sensitive to the change of mortality in voriconazole-treated and isavuconazole-treated patients with IA in the model of isavuconazole. In addition, changes in other parameters (parameters of the unit price of oral or intravenous isavuconazole, LY, QALY, etc.) did not result in an ICER above the $21,000 threshold. In the model of posaconazole, negative ICERs on the graph indicated a dominance of posaconazole over voriconazole. The reversal of results occurred when mortality decreased by 5% for posaconazole-treated with IA or increased by 5% for voriconazole-treated with IA.

### 3.3 Probabilistic sensitivity analysis

**Figure 3:**
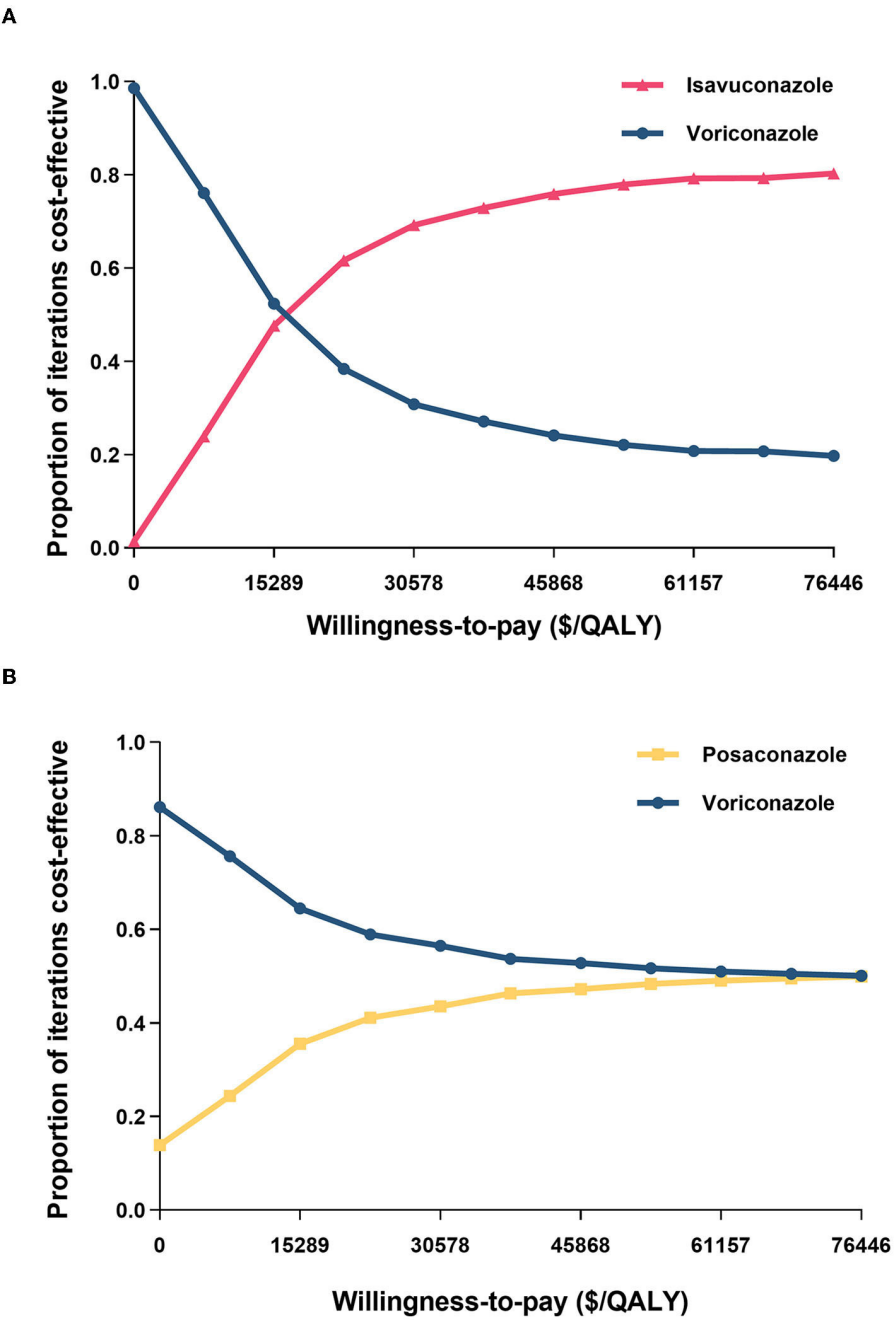
Cost-effectiveness acceptability curves of **(A)** isavuconazole versus voriconazole and **(B)** posaconazole versus voriconazole. QALY, quality-adjusted life year.

The PSA results revealed that the probability of voriconazole being cost-effective at a lower WTP threshold was higher than isavuconazole (Figure 3). Nevertheless, beyond the WTP threshold of $15,289 per QALY, the probability of isavuconazole was more cost-effective than voriconazole. Especially, isavuconazole was the optimal antifungal regimen in 61.6, 72.9, and 80.3% of simulations at the $22,934, $38,223, and $76,446 threshold, respectively.

### 3.4 Scenario analysis

The results of scenario analysis were outlined in Table 3. If the health insurance paid the voriconazole cost, ICER might increase to $27,705.55, but isavuconazole still had an advantage. The higher (14%) or lower (2%) detection rate of *mucorales* also did not affect the results of the base-case model. Scenario analysis considering price decreases after the loss of exclusivity revealed that posaconazole began to show a cost-effectiveness advantage when the unit price decreased by 15%. Once it fell to 30%, it would become a dominant strategy.

**Table 3:** Scenario analysis results.

**Scenario**	**Δ Cost ($)**	**Δ LY**	**Δ QALY**	**ICER ($/QALY)**
**BC: Isavuconazole–Voriconazole**	4,857.71	0.38	0.31	15,702.46
Payment of voriconazole by the health insurance	8,588.72	0.38	0.31	27,705.55
The prevalence of mucormycosis is 2%	4,882.06	0.39	0.32	15,256.44
The prevalence of mucormycosis is 14%	4,831.68	0.43	0.35	13,804.80
**BC: Posaconazole–Voriconazole**	2,111.92	0.04	0.03	64,466.57
Posaconazole after 15% price reduction	1,143.91	0.04	0.03	38,130.33
Posaconazole after 30% price reduction	175.91	0.04	0.03	5,863.67
The prevalence of mucormycosis is 14%	1,950.82	0.13	0.11	17,734.73

BC, base case value; LY, life year; QALY, quality-adjusted life year; ICER, incremental cost-effectiveness ratio.

## 4 Discussion

This study represented the inaugural exploration of the cost-effectiveness of three triazole antifungal agents for treating IMD. Its findings served to inform healthcare decision-making and aid in the allocation of resource with the goal of enhancing patient outcomes and medical efficiency.

Early definitive diagnosis, as well as the prompt initiation of antifungal therapy, are essential for the effective management of IMD. Immunocompromised patients, particularly those with hematologic malignancies undergoing chemotherapy, are at an increased risk of developing IMD, even in the absence of microbiological culture results (30). Initial antifungal therapy promptly is crucial for various reasons. These include the challenge of clinically distinguishing between IA and mucormycosis, both of which present with similar clinical presentations, as well as the rapid and aggressive disease progression associated with a high mortality rate (21, 31). Additionally, it is important to note that voriconazole lacks antifungal activity against *mucorales*. Any delay in commencing antifungal treatment significantly raises the mortality rate (32, 33). Therefore, the selection of a cost-effective treatment option is essential for optimal patient management.

Our base-case analysis indicates that, when compared to voriconazole, isavuconazole is a cost-effective option for suspected IMD, while posaconazole is not. This finding is attributable to the favorable outcomes of isavuconazole not only well-against *aspergillus*, but also *mucorales* (19). Although isavuconazole carries a high drug cost, its lower expenses in laboratory analysis (TDM), adverse events, and other treatment-related costs offset the high drug cost. On the other hand, posaconazole is effective against mucormycosis (34), but its drug cost is higher than that of voriconazole. Moreover, the costs associated with disease treatment do not differ significantly between posaconazole and voriconazole, further contributing to the disadvantage of posaconazole in terms of the total cost.

The one-way sensitivity analysis underscores the robustness of our findings. Consistent with previous studies in European countries, the model is highly sensitive to the mortality rate of patients with IA (15, 16, 18). The model also demonstrates that the prices of isavuconazole and posaconazole impacted its sensitivity, which is unsurprising. These brand drugs have not been on the Chinese market for long and are expensive under patent protection. However, as their patents expire, the introduction of generic drugs may lower their cost. Subsequent price reductions could affect the cost-effectiveness of these drugs differently. To explore this possibility, we performed a scenario analysis, which showed that a modest 15% decrease in the price of posaconazole could lead to a pharmacoeconomic advantage.

Our decision analytic model offered several advantages. Notably, it was constructed to encompass the entire treatment process for IMD in high-risk patients, encompassing empirical therapy prior to pathogen differential diagnosis and targeted therapy post-identification. This design closely mirrors the real-world diagnostic and therapeutic procedures. Furthermore, our model accommodated transitions between different treatment regimens by capturing the impact of various treatment choices through comprehensive data. This data included the proportion of oral and intravenous medications prescribed, diverse oral sequential therapy plans, and the cost of outpatient follow-up for antifungal treatment. This wealth of information provides healthcare professionals with a holistic perspective when making treatment decisions.

Despite its strengths, our study has several limitations. Firstly, like many modeling studies, the quality of available data limited the model inputs. The mortality data used in our model were derived from patients with hematologic malignancies and hematopoietic stem cell transplantation, which restricted the generalization of our results to other patient populations with varying risks of IMD. Additionally, due to the lack of epidemiological data on mucormycosis in high-risk patients with IMD in China, we relied on European data (15, 18). To address this issue, we simulated a broader range (2–14%) of mucormycosis prevalence in the scenario analysis. The results showed that at a 14% incidence rate, the ICER of posaconazole decreased to $17,734.73/QALY, falling below the WTP threshold ($38,223/QALY). Compared with voriconazole, posaconazole became more cost-effective. Secondly, since the absence of relevant studies exploring the impact of IMD on quality of life, our research did not set utility parameters based on this aspect. Finally, our study did not consider the drug-drug interactions and the direct or indirect costs associated with such interactions. It may underestimate the superiority of isavuconazole because of its lower frequency of drug-drug interactions (35).

## 5 Conclusion

Patients with a high risk of suspected IMD should be treated with antifungal therapy as soon as possible. When making treatment decisions, healthcare professionals should take into account the possibility and incidence of mucormycosis, as well as the effectiveness and safety of triazole antifungal drugs. Our results suggested that isavuconazole represented a cost-effective initial option for treating IMD in high-risk hematological patients prior to the differential diagnosis of pathogens. It would also be economical when a 15% reduction in posaconazole cost was achieved.

## Data availability statement

The raw data supporting the conclusions of this article will be made available by the authors, without undue reservation.

## Author contributions

GH: Formal analysis, Writing – original draft. QX: Formal analysis, Writing – review & editing. QL: Writing – review & editing. XL: Conceptualization, Methodology, Writing – review & editing. XS: Conceptualization, Methodology, Project administration, Writing – original draft.
